# Benign subglottic stenosis from a rheumatologist’ perspective: a narrative review

**DOI:** 10.1007/s10067-025-07681-9

**Published:** 2025-10-08

**Authors:** Louise C. Oskam, Jimmie Honings, Jolique A. van Ipenburg, Irene E. van der Horst-Bruinsma, Alexander H. Gelbard, Sander I. van Leuven, Henri M. Marres

**Affiliations:** 1 Department of Rheumatology, Radboud University Medical Center (Radboudumc), Nijmegen, The Netherlands; 2 Department of Otorhinolaryngology - Head and Neck Surgery, Radboud University Medical Center (Radboudumc), Nijmegen, The Netherlands; 3 Department of Pathology, Radboud University Medical Center (Radboudumc), Nijmegen, The Netherlands; 4 Department of Otolaryngology - Head and Neck Surgery, Vanderbilt University Medical Center, Nashville, TN USA

**Keywords:** Female preponderance, Immunosuppressive therapy, Rare disease, Subglottic stenosis, Unmet needs

## Abstract

Subglottic stenosis (SGS) is an umbrella term referring to a collection of rare diseases resulting in narrowing of the proximal airway directly below the glottis. SGS can follow iatrogenic injury (e.g., endotracheal intubation), can occur without antecedent injury (idiopathic SGS: iSGS), and can accompany autoimmune disease (e.g., Granulomatosis with Polyangiitis: GPA, Relapsing Polychondritis: RP). SGS is life-altering and life-threatening. Proximal airway obstruction generates dyspnea, limits exercise tolerance, and negatively impacts voicing. Taken together, SGS significantly reduces quality of life. Given its rarity, the diagnosis of SGS is often delayed. Fortunately, advances in our understanding of SGS have grown rapidly in recent years, aided by the widespread use of clinical testing. Useful diagnostic tools include pulmonary function testing, flexible endoscopy, computed tomography, laboratory testing, and pathology results. Treatment options are dependent on the underlying disease etiology but frequently involve endoscopic dilation. Especially in iSGS, invasive surgical options (cricotracheal resection (CTR)) are reserved for specific surgical candidates. While CTR can provide durable benefit, it has a significant risk profile and is not always curative. Alternative treatments which limit recurrent obstructive scar and decrease the need for repeated dilations are critical goals of the iSGS patient community. Although there is an established role for immunosuppressive agents in GPA and RP, solid proof of efficacy for immunosuppressive treatment in iSGS is lacking. New approaches have begun to investigate the role of adjuvant therapy in this patient subgroup. This article provides rheumatologists with the latest insights on the etiology, pathophysiology, diagnostic evaluation, and treatment of SGS.

## Introduction

**Fig. 1:**
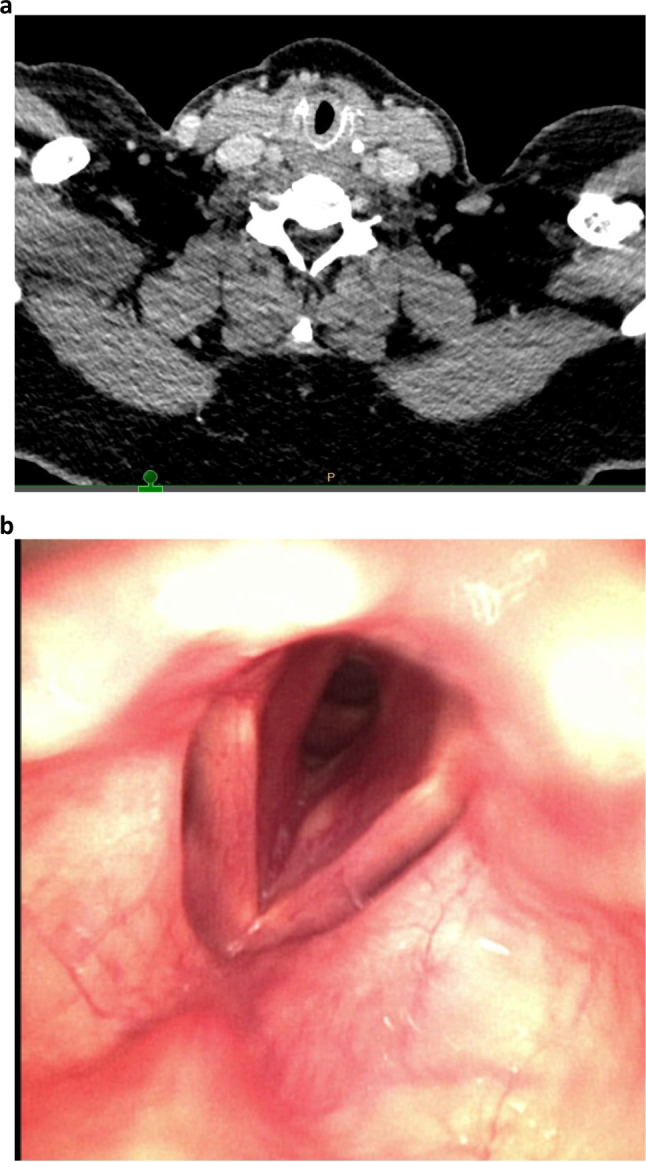
Computed tomography (CT) scan (**a**) and flexible endoscopy (**b**) in a 54-year-old female patient with a nine-month history of exertional shortness of breath and stridor. **a** Circumferential scar tissue directly under the true vocal cords. **b** Subglottic stenosis with obstruction of approximately 50%

Benign subglottic stenosis (SGS) is a rare phenomenon (estimated incidence of 0.2 per 100,000) due to obstruction of the central airway, directly below the vocal folds at the level of the cricoid cartilage (Fig. 1), resulting in severe dyspnea on exertion with an inspiratory stridor. Because of the rareness of the condition, diagnosis is often delayed by several years after the start of the first symptoms [1–3].

SGS can cause significant morbidity depending on the underlying cause. It can be the first sign of a systemic autoimmune disease such as granulomatosis with polyangiitis (GPA) or relapsing polychondritis (RP) or it can present during a flare of a previously diagnosed systemic autoimmune disease. Literature on idiopathic SGS (iSGS) has grown rapidly in recent years, and there is now a growing body of evidence that iSGS is also an inflammatory disorder as opposed to an isolated rim of scar tissue [4–8].

It is imperative that rheumatologists and internists are aware of SGS as a manifestation of systemic autoimmune disease and that they are involved in the diagnostic process and when applicable treatment of patients with SGS. Timely diagnosis is crucial to prevent unnecessary diagnostic procedures and delay in starting (immunosuppressive) treatment before irreversible damage to tissues occurs. The purpose of this article is to review the latest insights in etiology, pathophysiology, diagnostic evaluation, and treatment of benign SGS and to have a glimpse toward the future of this underrecognized condition.

## Etiology and presentation

Several causes of SGS have been proposed in the literature (Table 1). The most common cause of SGS is iatrogenic (post-intubation stenosis or injury after tracheostomy), with an estimated annual incidence of about 1 in 200,000 adults [9]. In a retrospective cohort study of patients with SGS, 54.7% were iatrogenic, 18.5% idiopathic, 18.5% autoimmune, and 8% traumatic. Patients with iatrogenic causes tend to have a more even sex distribution and higher rates of comorbidity (e.g., cardiovascular disease and diabetes mellitus) as opposed to patients with iSGS [10]. In a recent study, 60% of patients with laryngotracheal stenosis were classified as iatrogenic, 19% as idiopathic, and 8.4% as autoimmune [11]. In our experience, iSGS is nowadays more frequent than iatrogenic SGS, due to the use of high-compliant low-pressure cuffs and increased knowledge about the risks of tube diameter and (prolonged) intubation. Traumatic and long-lasting intubation are still risk factors for iatrogenic SGS. In this article, we will mainly focus on autoimmune SGS and iSGS.

**Table 1:** Etiology of acquired subglottic stenosis in adults

Trauma: - Internal/iatrogenic: intubation or tracheostomy trauma - External: blunt or penetrating trauma, foreign body aspiration, inhalational injury
Systemic autoimmune diseases: - Vasculitis: granulomatosis with polyangiitis, microscopic polyangiitis, eosinophilic granulomatosis with polyangiitis, Behçet syndrome - Relapsing polychondritis - IgG4-related disease - Sarcoidosis - Inflammatory bowel disease
Infection: - Mycobacterial - Fungal: Aspergillus, Histoplasma - Viral and bacterial: Herpes simplex, Staphylococcus aureus etc
Other: - Idiopathic - Amyloidosis - Radiation therapy

### Traumatic causes

**Fig. 2:**
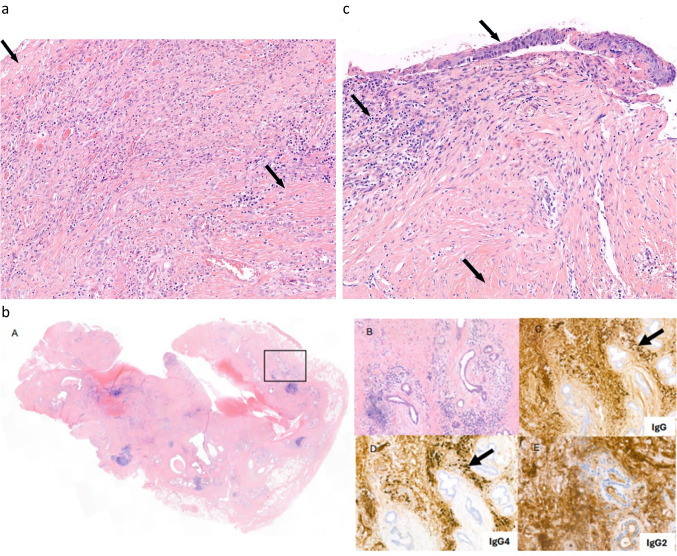
Pathology of subglottic stenosis of different etiology. **a** Tracheal resection with ulceration (left, arrow) and scarring (lower right, arrow) with inflammation in a patient with post tracheostomy stenosis, H&E staining, magnification 10/41×. **b** H&E staining (A) with trachea with chronic fibrosing inflammation with ulcerative aspect in a patient with Immunoglobulin 4-related disease. There is abundant fibrotic tissue in the substantia propria with scattered lymphoid tissue (B) with multiple plasma cells and scant eosinophilic cells. The plasma cells reveal expression of IgG (C) and IgG4 (D) (IgG4/IgG ratio 0.53), with only background staining in the IgG2 staining (E). Magnification 10/40×. **c** Cricotracheal resection with reactive changes of the squamous epithelium (upper arrow), chronic inflammation (middle arrow) and fibrotic tissue with reactive changes (lower arrow) in a patient with idiopathic subglottic stenosis. No abundant presence of plasma cells and/or eosinophilic granulocytes. No storiform fibrosis. H&E staining, magnification 10/41×

SGS after intubation is most often due to pressure-induced tissue ischemia and necrosis from the endotracheal or tracheostoma cuff or tube, leading to mucositis, perichondritis, and subsequently to subglottic scarring and fibrosis (Fig. 2a). Patients with internal trauma after intubation classically present with chronic progressive symptoms after 3 to 6 weeks of removal of the endotracheal tube. Computed tomography and endoscopy show nonspecific fibrotic thickening due to submucosal injury. Biopsy taking is not necessary, but can be performed to exclude other causes of the stenosis. In post-tracheostomy tracheal stenosis, scarring of the anterior tracheal wall results in a local collapse, similar to tracheomalacia, which produces a typical A-shaped trachea on computed tomography at the previous tracheostomy site. This type of stenosis can only be treated by resecting the affected airway segment.

### Systemic autoimmune disease

Various systemic autoimmune diseases can manifest as SGS; the two most common autoimmune diseases presenting with SGS are GPA and RP [12].

Patients with GPA-associated SGS most commonly present with airway symptoms such as chronic hemoptysis or acute severe dyspnea. SGS is estimated to occur in 10% of patients with GPA and can even present as an isolated manifestation without disease activity in other organs [13]. Although laryngeal manifestations of GPA are often described as a subglottic disease presenting with respiratory symptoms, subsite analysis shows that only 25% of GPA patients with laryngeal pathology had subglottic disease alone, with similar rates of glottic disease alone [14]. Patients with GPA and SGS were more likely to be female, younger at the time of diagnosis (36 vs. 49 years) and to have saddle-nose deformities, but were less likely to have renal involvement or to have PR3-ANCA positivity [13, 15]. Endoscopy shows an SGS with ulcerations, or mature and non-inflamed scar tissue when inflammation has waned. On computed tomography, GPA-related stenoses exhibit circumferential subglottic narrowing in 85% of cases, without calcifications [12]. Biopsy is required to confirm the diagnosis of vasculitis but has a lower diagnostic yield compared to other GPA subsites. Histology shows the presence of granuloma with necrosis or nonspecific inflammatory tissue [12]. ANCA negativity and biopsy negative findings do not necessarily exclude GPA [16].

Patients with RP have chronic or recurrent acute symptoms. They can have recurrent inflammation of cartilage of ears and/or nose (chondritis). The nature of airway problems is diverse, with tracheomalacia being the most common [17]. In a prospective observational cohort study, Ferrada et al. identified three subgroups of patients with RP. Subgroup 1 (type 1, 14%) of RP consists of patients with ear chondritis (100%), tracheomalacia (100%), saddle-nose deformity (90%), and SGS (80%). In this subgroup, 90% of patients are female and the median age at diagnosis is 39 years [18]. Computed tomography may show thickened tracheal rings and loss of cartilaginous support of the trachea, whereas endoscopy shows inflammation, narrowing, old scarring, and airway collapse due to tracheomalacia. Stenoses come with anterior involvement in 76%, with calcifications in 62%, and with extension to bronchi in 86% [12]. Tissue biopsy is not needed if diagnosis is clear from organ involvement and/or a biopsy elsewhere. Histology shows nonspecific inflammatory tissue.

Laryngeal involvement of immunoglobulin G4-related disease (IgG4-related disease) is a very rare manifestation, but is becoming increasingly recognized [19]. Patients with IgG4-related disease can present with chronic pulmonary complaints due to laryngopharyngeal involvement including SGS, lymphadenopathy, pancreatitis or cholangitis, among other presentations [20]. The serum IgG4 level can be raised but this is not a prerequisite. Tissue biopsy may show lymphoplasmacytic infiltrates enriched in IgG4-positive plasma cells with abundant storiform fibrosis (Fig. 2b) as well as (obliterative) phlebitis and a mild to moderate eosinophilic infiltrate, with striking similarities across the tissue of all different organs that may be involved although there are subtle variations. However, a prominent neutrophilic infiltrate in the absence of ulceration and/or presence of granulomas is relatively inconsistent with the diagnosis [21]. Moreover, in the late phase of organ involvement, fibrosis may predominate, with fewer plasma cells present, rendering the diagnosis of IgG4-related disease more difficult to make [22, 23]. Diagnosis of IgG4-related disease is based on a combination of clinical, biochemical, and histopathological results, as elevated concentrations of IgG4 in tissue and serum itself are not diagnostic for IgG4-related disease [22]. In addition, performing IgG2 immunohistochemistry may be helpful in rendering the diagnosis as, compared to non-IgG4-related diseases, a significantly lower tissue IgG2/IgG4 and IgG2/IgG ratio was found in IgG4-related disease [24].

### Idiopathic subglottic stenosis

ISGS is a rare chronic disease of unclear etiology, in the absence of any preceding injury, trauma, or underlying illness. It is characterized by a circumferential ring of fibrotic tissue due to hypertrophic scar formation at the level of the cricoid, which can result in severe exertional dyspnea and inspiratory stridor, among other respiratory symptoms, and is potentially life-threatening (Fig. 2c). The hypertrophic scar formation is considered to be the result of abnormal and dysregulated wound healing mechanisms [25]. ISGS presents with a greater degree of stenosis at the time of endoscopic dilation than GPA-related SGS [26].

A retrospective study with iSGS patients in the USA found an estimated annual incidence of 1:400,000. The vast majority of patients were female (99.1%) with a mean age at presentation of 51.2 years [27]. Similar results were found in Norway, and the mean interval between symptom onset and diagnosis was 3.1 years [2]. Another retrospective study of patients with iSGS patients in Canada found a mean annual incidence rate of 1:140,000 per year. There was a three-fold higher annual incidence compared to the abovementioned studies, demonstrating the highest rates of disease reported worldwide. Similarly, the majority of patients were female (95%) with a mean age at diagnosis of 47.8 years. The high rate of disease is most likely an intrinsic factor to the population, such as genetic background, socioeconomic factors, and/or environmental agents [28]. A study of the North American Airway Collaborative (NoAAC) revealed that iSGS patients are almost exclusively female (98.5%) with a mean age of 50 years. [29] In addition, 95% of patients with iSGS in this study were Caucasian; however, in California, USA, 14% of patients with iSGS were non-Caucasian [30].

A few cases are reported in the literature suggesting a genetic predisposition for iSGS. Dumoulin et al. describe two pairs of sisters as well as a mother and daughter presenting with iSGS. All known causes of subglottic stenosis were excluded but unfortunately, genetic examination was not performed [31]. Subsequently, Abbasi Dezfouli et al. described two monozygotic twin sisters with iSGS [32], which is in line with the findings of Hintze et al. who identified two sisters presenting with the condition [33]. Similarly, Drake et al. performed a retrospective observational study. They identified 810 patients with iSGS. A positive family history was reported in 44 patients in 20 families, quantifying the rate of familial clustering at 2.5% [34]. A high-resolution HLA typing (class I and II) of iSGS patients did not result in a detectable HLA association, suggesting that if genetic susceptibility exists, it likely lies outside the HLA locus [35]. Sharif et al. performed a query on the Online Mendelian Inheritance in Man (OMIM) database for single gene variants associated with a SGS phenotype. They identified and provided biologic context for 20 genes associated with fibrotic disease of the proximal airway, such as Filamin A, alpha (FLNA), Filamin B (FLN), and Latent TGFB binding protein-3 (LTBP3) [36].

Diagnosis of iSGS is often delayed for 1 to 2.5 years after the first symptoms occur because of the nonspecific respiratory symptoms and the rarity of the disorder. It is imperative to exclude other conditions before making a diagnosis of iSGS, because for some of these etiologies, effective medical treatment regimens are needed and available.

## Diagnostic evaluation

Diagnostic guidelines for patients with SGS are not available. In our opinion, the diagnostic work-up of SGS should consist of a basic set of examinations, including thorough history taking, physical examination, laboratory testing, pulmonary function testing, radiological and endoscopic examination, and preferably, tissue biopsy (Table 2).

**Table 2:** Diagnostic evaluation of patients with subglottic stenosis

History and physical examination	Prior intubation, tracheostomy, neck trauma, radiation Known autoimmune disease Skin- and/or joint complaints Constitutional symptoms
Laboratory examination	ESR, CRP, kidney function, liver enzymes, hemocytometry, ANCA, IgG4, urinalysis On indication: IgM RF, anti-CCP, ANA, ACE, M-protein, free chain ratio
Microbiology	On indication: culture of biopsies, culture of washing, tuberculosis testing
Pathology	Biopsies of SGS or tracheal resection for histology: granuloma, vasculitis, IgG4-related disease, malignancy
Radiology/nuclear medicine	Computed tomography of neck and chest for anatomical characterization of subglottic scar On indication: magnetic resonance imaging of trachea for evaluating soft tissue and distinguish inflammation from fibrosis On indication: positron emission tomography for detecting active inflammation and cartilage involvement and to rule out systemic disease
Pulmonary function testing	Spirometry with flow volume curve for detecting fixed obstruction and diminished in- and expiratory flow
Endoscopy	Laryngoscopy or tracheobronchoscopy to characterize and measure stenosis

Abbreviations: ESR, erythrocyte sedimentation rate; CRP, C-reactive protein; ANA, antinuclear antibodies; ANCA, antineutrophil cytoplasmic autoantibodies; IgM RF, IgM rheumatoid factor; anti-CCP, anti-citrullinated peptides; ACE, angiotensin-converting enzyme; IgG4, immunoglobulin 4

Differentiating iSGS from autoimmune SGS can be difficult, especially in the later stages of autoimmune SGS, when acute inflammation can be vanished. Whereas in iSGS, laryngoscopy or tracheobronchoscopy often show only a ring of scar tissue, in GPA, it may show ulcerative lesions and inflammatory pseudotumors.

A positive ANCA is particularly helpful in differentiating between iSGS and GPA-associated SGS as it is the only significant positive serological testing in SGS patients [37].

Furthermore, histology is needed in the diagnostic process to possibly confirm autoimmune disorders and to rule out rare and serious causes. In iSGS patients, often only a fibrotic lesion or aspecific inflammation is demonstrated in biopsies.

Pulmonary function testing in patients with SGS shows a fixed in- and expiratory obstruction, resulting in a diminished in- and expiratory flow. Peak expiratory flow (PEF) has been correlated with Cotton-Myer grade of stenosis and, in the follow-up of patients with SGS, can be monitored at home using an inexpensive device [38].

Axial computed tomography imaging is essential in the anatomic characterization of subglottic scar. Together with PEF, computed tomography is recently recognized as clinical end points in future clinical trial design [39].

## Pathophysiology of subglottic stenosis

In the comprehensive review of Marchioni et al., the wound healing process in the trachea is extensively described, consisting of four programmed phases: hemostasis, inflammation, proliferation, and maturation/remodeling. Important players in the wound healing process are the epithelium, subendothelial extracellular matrix, chemokines, neutrophils, monocytes, macrophages, cytokines, growth factors, and fibroblasts. Any deviation from the four programmed phases of wound healing can lead to dysfunctional wound repair, excessive accumulation of extracellular matrix, and ultimately tissue fibrosis. Its round shape and cicatricial scar formation render the trachea particularly susceptible to fibrotic stenosis [40].

In iatrogenic SGS, upper airway mucosal injury triggers an inflammatory response that results in upregulation of cytokines such as IL-1α, IL-6, IL-16, tumor necrosis factor α (TNF-a), fibroblast growth factor (FGF), and platelet-derived growth factor (PDGF), promoting fibroblast activation. The adaptive immune system, as well as dysregulated macrophages, seems to have a central role in granulation and hypertrophic scarring after upper airway mucosal injury [8, 40–42].

In iSGS, a different immunological derangement may be at the basis of the inflammatory response. Histology shows pronounced subepithelial fibrosis and collagen remodeling resulting in an intraluminal scar centered at the level of the cricoid. Biopsies of subglottic scar tissue show activated macrophages and Th2 cells, possibly leading to excessive deposition of extracellular matrix [6–8]. In addition, biopsies demonstrate upregulation of the IL-17A/IL-23 inflammatory axis [5]. A laboratory study on fibroblast cell lines from iSGS subjects, idiopathic pulmonary fibrosis subjects, and normal control airways demonstrated that IL-17A directly drives iSGS scar fibroblast proliferation, synergizes with transforming growth factor ß1 to promote extracellular matrix production, and amplifies local inflammatory signaling [43]. A role for IL-17A has also been increasingly recognized in autoimmune disorders such as GPA [14]. Furthermore, the innate immune system seems to play a role in iSGS by the displacement of native microbiome into the lamina propria via an abnormal epithelial barrier resulting in an inflammatory insult [44–46]. In a recent study by So et al., flow cytometry and single-cell RNA sequencing of scar tissue of iSGS patients demonstrates increased fibroblast, endothelial, and inflammatory cell types but a decreased epithelium compared with normal tissue of these patients [47].

ISGS predominantly affects fertile and perimenopausal women. A retrospective analysis of surgical specimens from these patients showed an imbalance between estrogen-alpha, estrogen-beta, and progesterone receptors. This hormonal background may be involved in inappropriate inflammation and increased stenosis susceptibility [48]. Another study confirms the presence of estrogen receptors in the subglottic area. Increased expression of estrogen-alpha in the epithelium and estrogen-beta in glands and ducts in iSGS compared to controls may help to explain the predisposition to stenosis in young women. [49] In addition, overexpression of progesterone receptors drives the iSGS pathogenesis [50]. Menopausal changes seem to play a role in stenosis grade, receptor patterns, and in recurrence rate, and birth control possibly provides a protective effect [48, 51]. Yet, tissue of patients with iSGS does not reveal estrogen or progesterone receptor overexpression in the cells [52].

## Treatment

Regardless of the underlying cause, local treatment options of SGS consist of endoscopic incision and dilatation with or without adjuvant medical therapy, endoscopic or awake intralesional injection with corticosteroids, and cricotracheal resection (CTR). In case of autoimmune disease, systemic treatment is indicated. Currently, recommendations or guidelines on treatment are not available, and there is considerable heterogeneity in management strategies [53].

### Endoscopic surgical treatment

Endoscopic surgery for SGS is a critical aspect of patient management enabling short-term symptomatic relief. Endoscopic techniques include dilation with either bougies or a balloon, radial incisions or scar resection with carbon dioxide laser or cold knife, and combinations of these techniques. Adjunctive measures include local glucocorticoid injections. A retrospective study from Feinstein et al. [54] demonstrated that in patients with SGS due to iatrogenic, autoimmune, or idiopathic cause(s), neither surgical technique nor grade of stenosis was related to the surgical intervals. Unfortunately, SGS recurs in almost all patients with SGS treated with endoscopic surgery. The interval varies widely between patients, between months, and many years.

In patients with GPA and RP, improvement rates after dilatation and submucosal corticosteroid injection are higher than in patients with idiopathic and traumatic SGS [55]. Endoscopic CO2 laser excision is a safe and effective local intervention for GPA-associated SGS [56].

In case of iSGS, a multitude of endoscopic techniques are being used. In the review of Lavrysen et al., no superior modality could be identified [57]. Unfortunately, the recurrence rate of narrowing of the trachea after endoscopic dilation in these patients is high (50% of patients needs a recurrent endoscopic dilatation during a 5-year follow-up period) [58]. Methods to determine optimal timing for the next intervention are warranted [59]. Patients with iSGS who were completely or partially compliant to triple therapy (inhaled corticosteroid, proton pump inhibitor (PPI) and antibiotic (trimethoprim-sulfamethoxazole)) appear to demonstrate increased intervals prior to recurrence [60].

### Awake office-based serial intralesional steroid injection

Franco et al. demonstrated that in-office serial intralesional steroid injection (SILSI) in the awake outpatient setting can improve the airway caliber in iSGS and is equivalent to endoscopic surgical treatment. As the underlying mechanism, they suggest that iSGS can be viewed as a chronic scarring/inflammatory condition that can benefit from steroid scar-modification therapy as a pharmacological approach [61]. In the review of Luke et al., SILSI is demonstrated as a potential treatment option for patients with traumatic SGS, autoimmune SGS, or iSGS, significantly lengthening the surgery-free interval and potentially reducing surgical burden. The injection was found to be safe with few reported side effects [62]. In the retrospective case series of Neevel et al., 91% (10/11) of patients with SGS of various causes who initiated SILSI had a mean increase in the surgery-free interval of 4.6 months. However, in the study of Hoffman et al., SILSI did not significantly lengthen the time to recurrence [63]. Until today, SILSI has never been compared to placebo in a randomized trial.

Systemic side effects of glucocorticoids occurred in 32% of patients after initiating SILSI [64]. Systemic absorption of steroid following SILSI occurs with acute hypothalamic–pituitary–adrenal (HPA) axis suppression; however, normalization of HPA axis function by day 7 suggests no cumulative systemic steroid side effect with serial injections [65]. Clinicians should make their patients aware of the most frequent side effects, and special attention should be given to women of reproductive age to inform them of the possibility of menstrual irregularities during SILSI [66].

Furthermore, in-office steroid injections could offer cost savings for the patient according to a retrospective study and cost analysis of O’Dell et al. [67].

### Surgery

CTR with thyrotracheal anastomosis is an open surgical approach for adult patients with iSGS and other causes of SGS that yields the only possible definitive solution for SGS. Maurizi et al. found laryngotracheal resection for traumatic or iSGS a safe and effective therapy with a very high rate of success (98.7%) [68]. In a retrospective cohort study of 114 patients with traumatic or iSGS by Jethwa et al., 5% of patients required permanent tracheostomy. Factors predicting treatment failure include traumatic stenosis, longer T-tube duration (when needed), combined glottic/subglottic stenosis, start of stenosis at the level of vocal cords, postoperative minor complications, and need for repeat surgery [69].

Open airway reconstruction is much more effective in iSGS than in GPA-associated SGS as GPA SGS is associated with greater rates of tracheotomy [26]. However, a systematic review of Almuhanna et al. found excellent results of open transcervical approach in patients with GPA SGS, mainly after failure of other endoscopic techniques. Immunosuppressive treatment was essential for stabilizing the active disease [70].

In comparison with other treatment modalities, CTR maintained the lowest rate of recurrent operation (5%), followed by endoscopic resection with adjuvant medical therapy (30%) and endoscopic dilation (50%) in patients with iSGS in the NoAAC cohort [58]. Given the invasive nature and risk of complications, open surgery should be reserved for refractory cases of iSGS. Recurrence may occur > 10 years after open treatment, highlighting the importance of long-term follow-up. Patients should be counseled about the potential for worsening voice quality with the open approach [71].

A pilot study on endoscopic resection and mucosal reconstitution with epidermal grafting (the Maddern procedure) in patients with iSGS demonstrates durable improvement in peak expiratory flow rate and a low complication rate. The results of long-term follow-up are not yet available [72].

### Medication

As of yet, there is no evidence for any significant effect of immunosuppressive therapy in patients with traumatic SGS. A randomized clinical trial in Iran showed no significant effect of treatment with corticosteroids (prednisolone 15 mg once daily) in 50 patients with postintubation tracheal stenosis (10 patients with SGS) in lengthening the endoscopic dilatation intervals and in decreasing the number of patients needing airway resection [73].

In patients with autoimmune SGS, immunosuppressive therapy is obviously the cornerstone of treatment and is preferably initiated before endoscopic surgery. Multidisciplinary consultation with a rheumatologist or internist is for this reason imperative for an effective and safe treatment strategy.

Whereas the recently published European Alliance of Associations for Rheumatology (EULAR) recommendations for the management of ANCA-associated vasculitis provide clear guidelines on the treatment of systemic GPA, literature on systemic treatment in patients with GPA-associated SGS is scarce [74, 75]. In a recently published retrospective study in Minnesota, USA, a reduced risk of recurrence was observed in patients with GPA-associated SGS (limited GPA 60%, SGS as sole manifestation 80%) treated with maintenance therapy with rituximab (following initial endoscopic treatment) compared to treatment with non-rituximab agents (prednisone monotherapy, methotrexate, azathioprine) [56]. Three other retrospective studies demonstrate the combination of methotrexate and rituximab, leflunomide, and sirolimus (in addition to conventional medical and endoscopic treatment) as possible effective treatment options in prolonging dilatation intervals [76–78]. Fortunately, most recently, the British Society for Rheumatology Management recommendations for ANCA-associated vasculitis were published, including management of GPA-associated SGS. In patients with GPA-associated SGS, induction and maintenance therapy following the recommendations for systemic GPA treatment are recommended. Systemic therapy with cyclophosphamide or rituximab, in combination with glucocorticoids, provides early disease control and delays the need for recurrent dilations [79]. During follow-up, local activity of GPA-associated SGS can be assessed by history, laboratory testing (however, in ENT-localized GPA, CRP, and ANCA are often negative), measuring the PEF and repeated endoscopy. According to expert opinion, in the case of refractory disease, methotrexate can be prescribed, in addition to rituximab. In the case of remission, but progression of fibrotic scar, only endoscopic surgery can be indicated. Patients should be followed during the course of their immunosuppressive therapy (on average 24 months); further follow-up is based on the characteristics of the individual patient.

Treatment of RP is, due to the rarity of this disease, based on expert opinion. Following clinical guidelines, patients with tracheal involvement are treated with systemic glucocorticoids in combination with conventional or biologic Disease Modifying Anti-Rheumatic Drugs (DMARDs), such as methotrexate, azathioprine, infliximab, or tocilizumab. There is scarcity in high-quality evidence to guide in choosing between different agents [80].

SGS due to IgG4-related disease is a very rare phenomenon. Atienza-Mateo et al. described a case of isolated IgG4-related disease SGS, which was successfully treated with oral glucocorticoids and rituximab infusions [20]. Moreover, Maughan et al. describe rituximab as an effective treatment in patients with laryngeal IgG4-related disease [19].

Due to increased understanding of the pathophysiology of iSGS, there is now a growing body of evidence that systemic therapeutic interventions have a role in increasing the time to recurrence of the stenosis. Triple therapy with a PPI, inhaled corticosteroid, and an antibiotic (trimethoprim-sulfamethoxazole), as adjuvant therapy after endoscopic surgery, is possibly effective. The PPI prevents gastroesophageal reflux disease (GERD), although the evidence of the role of GERD in iSGS is mixed. Patients with iSGS and gastroesophageal reflux disease may experience some symptom benefit with PPI [81]. The inhaled corticosteroid has local anti-inflammatory effects; however, the amount of corticosteroid that actually deposits at the subglottis is likely to be small. The antibiotic trimethoprim-sulfamethoxazole may be helpful in eliminating pathogenic upper airway bacteria [81]. According to Hoffman et al., evidence of efficacy of adjuvant medications in prolonging time to recurrence is lacking [81]. However, a retrospective study of Bowen et al. did demonstrate an increased mean time of recurrence in iSGS patients who were completely or partially compliant with the aforementioned triple therapy [60]. A recent study performed a secondary analysis of the NoAAC in a cohort of iSGS patients showed a transient delay in recurrence among centers that routinely prescribed triple therapy to iSGS patients after endoscopic dilation, which was further supported by patient-reported data and peak expiratory flow data [82].

Besides triple therapy, treatment with immunosuppressive drugs is possibly effective in the treatment of iSGS. Recently, Awadallah et al. demonstrated in a retrospective cohort study in patients with highly recurrent iSGS treated with methotrexate and/or rituximab a trend toward longer intervals between endoscopic surgeries [83]. Very recently, seven perimenopausal patients with iSGS were treated with the mammalian target of rapamycin (mTOR) inhibitor everolimus 1.5 mg once daily for 42 days as an adjuvant after endoscopic surgery in a phase 1 nonrandomized clinical trial. Rapamycin is a macrocyclic antibiotic and acts as an inhibitor of mTOR. It has been reported to inhibit hepatic fibrosis, lung fibrosis, renal fibrosis, and subglottic stenosis. This effect may be due to its inhibition of fibroblast proliferation and collagen expression (84, 85). Patients sustained postdilation peak expiratory flow for 13 weeks [84]. In addition, a single-arm, open-label study on the effect of IL-17 inhibition by the biologic drug ixekizumab in decreasing scar fibroblast formation in patients with iSGS is currently ongoing at Yale University (registered ClinicalTrials.gov ID NCT05309616).

## Future perspectives

There is an unmet need in the treatment of patients with autoimmune SGS and especially in iSGS, because of the high rate of recurrence of stenosis after endoscopic surgery and the negative impact of CTR on voicing. Based on the recent publications on the pathophysiology of iSGS, there seems to be a role for immunosuppressive agents in the treatment strategy of these patients. As mentioned, the results of a recent phase 1 nonrandomized clinical trial on everolimus showed encouraging results [84]. Similarly, azithromycin is also a macrocyclic antibiotic with immunomodulatory effects in addition to antibacterial effects, reducing inflammatory cytokine production. A study of Ghaderi et al. demonstrates that azithromycin prevents pro-fibrotic gene expression and myofibroblast differentiation and can help protect mice from developing SGS [85].

In the past decade(s), the effectiveness and safety of conventional, biologic, and targeted synthetic DMARDs in the treatment of patients with autoimmune diseases are established. Randomized clinical trials with these immunosuppressive agents, with a sufficient follow-up, are needed in patients with autoimmune SGS and iSGS.

Pharmacological approaches based on antifibrotic medications—already approved for the treatment of idiopathic pulmonary fibrosis—are currently being tested in animal models. Both pirfenidone and nintedanib seem to be promising agents in the treatment of SGS [40]. These novel treatment strategies may enhance the therapeutic armamentarium of this underrecognized and difficult-to-treat condition.

## Conclusion

SGS is a life-altering and life-threatening condition. Given its rarity, the diagnosis is often delayed. Fortunately, advances in our understanding of SGS have grown rapidly in recent years, aided by widespread use of clinical testing. Treatment options are dependent on the underlying disease etiology but frequently involve endoscopic dilation. Although there is an established role for adjuvant medications including systemic immunosuppressive agents in GPA and RP, solid proof of efficacy for systemic immunosuppressive treatment in iSGS (or post-intubation SGS) is lacking. Yet, given the recalcitrant nature of the disease despite surgical management for some iSGS patients, new approaches have begun to investigate the role of adjuvant therapy in this patient subgroup.

## Data Availability

The authors declare they have no conflicts of interest.
